# *Sost* Haploinsufficiency Provokes Peracute Lethal Cardiac Tamponade without Rescuing the Osteopenia in a Mouse Model of Excess Glucocorticoids

**DOI:** 10.1016/j.ajpath.2018.12.007

**Published:** 2019-04

**Authors:** Behzad Javaheri, Eleanor Herbert, Mark Hopkinson, Ahmed Al-Jazzar, Andrew A. Pitsillides

**Affiliations:** ∗ Department of Comparative Biomedical Sciences, The Royal Veterinary College, London, United Kingdom; † Experimental Histopathology, The Francis Crick Institute, London, United Kingdom; ‡ College of Veterinary Medicine, King Faisal University, Al-Hofuf, Saudi Arabia

## Abstract

Glucocorticoid-induced secondary osteoporosis is the most predictable side effect of this anti-inflammatory. One of the main mechanisms by which glucocorticoids achieve such deleterious outcome in bone is by antagonizing Wnt/β-catenin signaling. Sclerostin, encoded by *Sost* gene, is the main negative regulator of the proformative and antiresorptive role of the Wnt signaling pathway in the skeleton. It was hypothesized that the partial inactivation of sclerostin function by genetic manipulation will rescue the osteopenia induced by high endogenous glucocorticoid levels. *Sost*-deficient mice were crossed with an established mouse model of excess glucocorticoids, and the effects on bone mass and structure were evaluated. *Sost* haploinsufficiency did not rescue the low bone mass induced by high glucocorticoids. Intriguingly, the critical manifestation of *Sost* deficiency combined with glucocorticoid excess was sporadic, sudden, unprovoked, and nonconvulsive death. Detailed histopathologic analysis in a wide range of tissues identified peracute hemopericardium and cardiac tamponade to be the cause. These preclinical studies reveal outcomes with direct relevance to ongoing clinical trials that explore the use of antisclerostin antibodies as a treatment for osteoporosis. They particularly highlight a potential for increased cardiovascular risk and may inform improved stratification of patients who might otherwise benefit from antisclerostin antibody treatment.

Glucocorticoids (GCs) are anti-inflammatory molecules synthesized and secreted by the adrenal glands that exert significant influence on the physiological functioning of several systems, including adaptation to stress, metabolism, and regulation of immune responses. The signaling axis of GCs consists of the hypothalamic–pituitary–adrenal axis influenced by many factors, including neuroinflammation, physical stress, circadian rhythm, and negative feedback. GCs are used in the treatment of various diseases, such as asthma, rheumatoid arthritis, and systemic lupus erythematosus^1^, ^2^, ^3^, ^4^ with many reported side effects.^5^, ^6^ Significantly, GC-induced osteoporosis (GIO) is the most predictable side effect and the commonest cause of secondary osteoporosis, leading to increased fracture risk in 30% to 50% of patients receiving GCs.^5^, ^6^, ^7^, ^8^, ^9^ Thus, there remains a significant unmet clinical need for the development of therapies to prevent and/or treat GIO.

The central feature in the pathogenesis of GIO is the suppression of bone formation. Previous studies suggest that GCs decrease the number and function of osteoblasts by a reduction in osteoblastogenesis and impairment in osteoblastic differentiation and maturation.^10^, ^11^ This consequently favors adipogenesis, most likely dictated by up-regulation of peroxisome proliferator–activated receptor γ 2, leading to increased bone marrow adiposity.^12^, ^13^, ^14^, ^15^ In addition, GCs decrease osteoblast viability and activity, ultimately leading to a reduction in bone mass and compromised bone structure.^11^ Osteocytes are the other key players in GIO because GCs induce their apoptosis.^16^, ^17^, ^18^ This is likely achieved by the GC-mediated disruption of the osteocyte–lacunar–canalicular network, essential for osteocyte viability and maintenance of the bone's material properties.^11^ These changes may explain the impairment of the biomechanical properties in the surrounding bone^16^ and may account for the loss of bone strength that occurs before the loss of bone mineral density.^19^

One of the mechanisms by which GCs drive bone loss is by inhibition of the Wnt/β-catenin signaling pathway,^15^, ^20^, ^21^, ^22^ critical for the differentiation of mesenchymal cells toward mature osteoblasts, bone formation, and mechanoadaptive responses.^11^, ^16^, ^23^, ^24^ A natural antagonist of Wnt signaling, sclerostin (*Sost* gene product), predominantly secreted by osteocytes, is a potent inhibitor of osteoblastic mineralization.^25^, ^26^, ^27^ Thus, its deficiency provokes marked increases in bone mass achieved by a range of targets,^28^, ^29^, ^30^, ^31^, ^32^, ^33^, ^34^, ^35^, ^36^, ^37^, ^38^ without any significant impact on osteocyte differentiation.^39^, ^40^

Several studies have reported contradictory results on the relationship between GC excess and sclerostin.^41^, ^42^, ^43^, ^44^, ^45^ Previous studies have reported that *in vivo* antisclerostin antibody treatment prevents the reduction in bone mass and strength induced by GC excess.^41^, ^42^ Despite these established links between sclerostin levels and the prevention of GC-induced changes in bone mass, there remains controversy about how these links affect patients, in which there have been discordant reports of decreased serum sclerostin in patients after 1 week of GC treatment and increased serum sclerostin at later time points.^43^ Similar disparities in the levels of sclerostin are also seen in GC-related disease states, in which both increased and decreased levels are reported in patients with excess GCs.^44^, ^45^ The reasons for these contradictory observations on the relationship between sclerostin and excess GCs are unclear, raising the question whether modulation of sclerostin function indeed counteracts the deleterious effect of endogenously raised GC levels on bone mass and strength.

We tested this possibility by determining whether genetically determined sclerostin deficiency was capable of rescuing the compromised bone mass that occurs with GC excess. To address this question, sclerostin-deficient mice were crossed with an established mouse model of GC excess because of an *N*-ethyl-*N*-nitrosourea–induced mutation in corticotropin-releasing hormone (*Crh*),^46^ and the effects on bone mass and structure were evaluated along with detailed histopathologic analysis in a wide range of tissues. Our preclinical findings are relevant to ongoing clinical trials in which serious fatal cardiovascular adverse events were reported in patients receiving antisclerostin antibody. They particularly highlight a potential need for patient stratification to help realize the potential benefit of such treatment.

## Materials and Methods

### Animals

Frozen sperm from a male *Sost* knockout (KO) mouse in the C57BL/6NTac background was purchased from the Knockout Mouse Project Repository at the University of California Davis, and used to fertilize ova from C57BL/6J wild-type (WT) mice as described previously^39^; hetero/haplozygosity was confirmed by genotyping. The mouse model for excessive circulating GC concentrations was a gift from Medical Research Council (Oxfordshire, UK) and was generated in C57BL/6J mice by an *N*-ethyl-*N*-nitrosourea–induced mutation in corticotropin-releasing hormone (*Crh*) at −120 bp of the promoter region, resulting in a gain-of-function mutation (*Crh*^+/−120^) and mated with C3H/HeH as described previously.^46^ Female *Sost* homozygous KO (*Sost*^−/−^) mice were crossed with male *Crh*^+/−120^ to produce *Sost*-*Crh*^+/−120^ (*Sost* heterozygous/*Crh* heterozygous) and *Sost*^+/−^ mice. Mice were housed in polypropylene cages under 12 hours light/dark cycle at 21 ± 2°C with free access to Rat/Mouse One maintenance diet (Special Diet Services, Witham, UK) and water ad libitum. The studies used only male mice throughout. All procedures complied with UK Animals (Scientific Procedures) Act 1986, were approved by the Royal Veterinary College's Ethics committee, and followed Animal Research: Reporting of In Vivo Experiments guidelines.^47^

### X-Ray MicroCT

*In vivo* scanning of the entire right tibia at 1 month of age under 2% isoflurane-induced anesthesia and postmortem at 2 months of age were achieved with the use of a Skyscan 1176 X-ray microcomputed tomography machine (Skyscan, Kontich, Belgium). The X-ray tube was operated at 40 kV, 600 μA, with a voxel size of 9 μm, an exposure of 2000 ms, and a rotation step of 0.800 degrees. The radiation dose from the microcomputed tomography (microCT) scanning was estimated to be approximately 500 mGy for each scan, which has been proved to cause no significant effect on bone adaptations.^48^ Slices were reconstructed with the use of NRecon1.6; trabecular and whole bone analyses were performed as described previously.^24^ For morphometric trabecular analysis appearance of the trabecular bridge that connected the two primary spongiosa bone islands was set as a reference point for analysis of proximal tibia metaphyseal trabecular bone; 5% of the total bone length from this point (toward diaphysis) was used. For cortical analysis, after segmentation, alignment, and removal of fibula a minimum threshold was used in Slice Geometry to calculate mass: cross-sectional area (CSA), mean cross-sectional thickness, second moment of area around minor axis, second moment of area around major axis, and predicted resistance to torsion along a central 70% portion of the entire tibia length excluding regions that contained trabecular bone.

### Histologic Analysis

Tibia, heart, lungs, liver, spleen, kidneys, and brain were fixed in 4% formaldehyde (from paraformaldehyde Alfa Aesar Inc., Ward Hill, MA) and stored in 70% ethanol before routine processing into paraffin and production of slides sectioned at 4 μm and stained with hematoxylin and eosin. Stained slides were evaluated by a board-certified veterinary pathologist (E.H., The Francis Crick Institute, London, UK).^49^ Total numbers of animals examined were *Sost*^+/+^ WT (*n* = 4), *Sost*^+/−^ heterozygous KO (*n* = 7), *Sost*^−/−^ homozygous KO (*n* = 4), *Crh*^+/+^ WT (*n* = 4), *Crh*^+/−120^ heterozygous KO (*n* = 4), and *Sost*-*Crh*^+/−120^ heterozygous (*n* = 5) mice. In addition, *Sost*-*Crh*^+/−120^ (*n* = 3) mice which spontaneously died were stored in 70% ethanol and examined histologically. Hearts from these animals were examined by using multiple step levels and extensive sectioning throughout the block.

### Statistical Analysis

Statistical analyses were performed with R, version 3.1.1 (R Foundation for Statistical Computing, Vienna, Austria; *http://www.r-project.org*, last accessed August 29, 2018). Measurements were summarized as means ± SEM. Linear model (two-way analysis of variance) was used to determine the effects of age (1 and 2 months) and genotype (*Sost* heterozygous and homozygous as well as *Crh* heterozygous and double *Sost*-*Crh* heterozygous and their corresponding WT littermates) and their interaction on all phenotypic measurements. Bonferroni *post hoc* correction was performed for whole bone measurements. The statistical significance level was set at 5%.

## Results

### *Sost* Haploinsufficiency Does Not Rescue Compromised Bone Mass Induced by Excessive GCs

**Figure 1:**
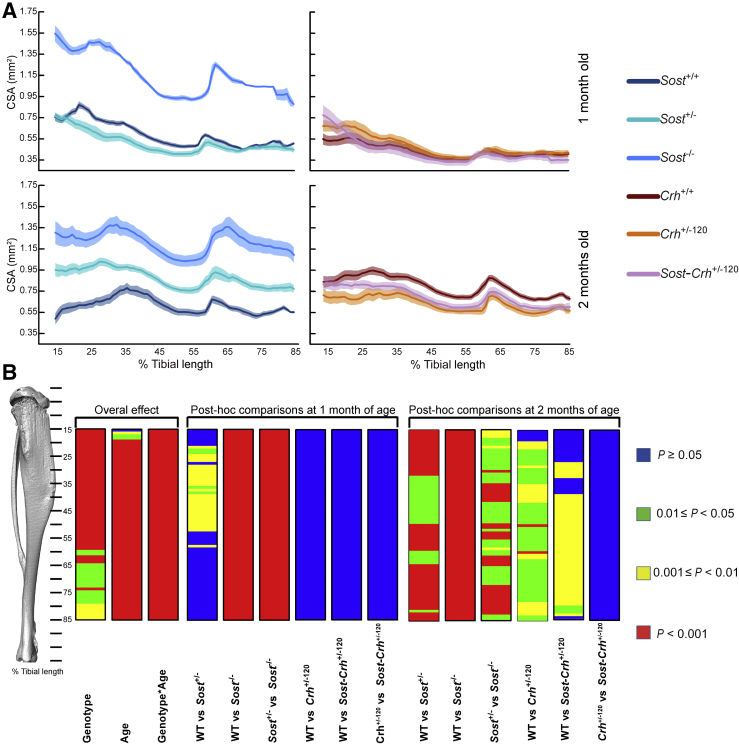
*Sost* haploinsufficiency does not rescue low bone cross-sectional area (CSA) induced by excessive glucocorticoids (GCs). **A:** Average CSA of *Sost* wild-type (WT) (*Sost*^+/+^), *Sost* heterozygous knockout (KO) (*Sost*^+/−^), *Sost* homozygous KO (*Sost*^−/−^), *Crh* WT (*Crh*^+/+^), *Crh* heterozygous (*Crh*^+/−120^), and double *Sost*-*Crh* heterozygous (*Sost*-*Crh*^+/−120^) mice at 1 and 2 months of age. Lines represent the means; shading, the SEM. **B:** Graphic heatmap representation of statistical significance of the effect of genotype, age, their interactions as well as *post hoc* analysis along a 15% to 85% portion of the whole tibia length, excluding proximal and distal metaphyseal bone. Line graphs represent means ± SEM (shadow). *n* = 8 mice per group.

The designed studies tested whether genetically determined *Sost* deficiency rescued low bone mass induced by excessive GCs *in vivo*. Trabecular bone was analyzed, and the entire tibial cortex was analyzed proximodistally in a nonbiased fashion at 1 and 2 months of age. Both age and genotype independently affected the cortical CSA, and significant interaction was evident between age and genotype along the entire tibial length (Figure 1). The detailed *post hoc* analyses at 1 month of age revealed, as expected, significantly higher CSA in *Sost* homozygous KO (*Sost*^−/−^) mice than in *Sost*^+/+^ WT mice (Figure 1) as well *Sost*^−/−^ mice compared with *Sost*^+/−^ mice along the entire tibia. Unexpectedly, lower cortical CSA was observed in restricted regions of the tibia in *Sost*^+/−^ mice compared with *Sost*^+/+^ WT littermates (Figure 1). At 2 months of age, CSA was significantly higher in *Sost*^+/−^ mice than in *Sost*^+/+^ WT mice between 15% and 85% of the length and also in *Sost*^−/−^ mice compared with *Sost*^+/+^ WT and *Sost*^+/−^ mice across the entire tibial cortex.

**Supplemental Figure S1:**
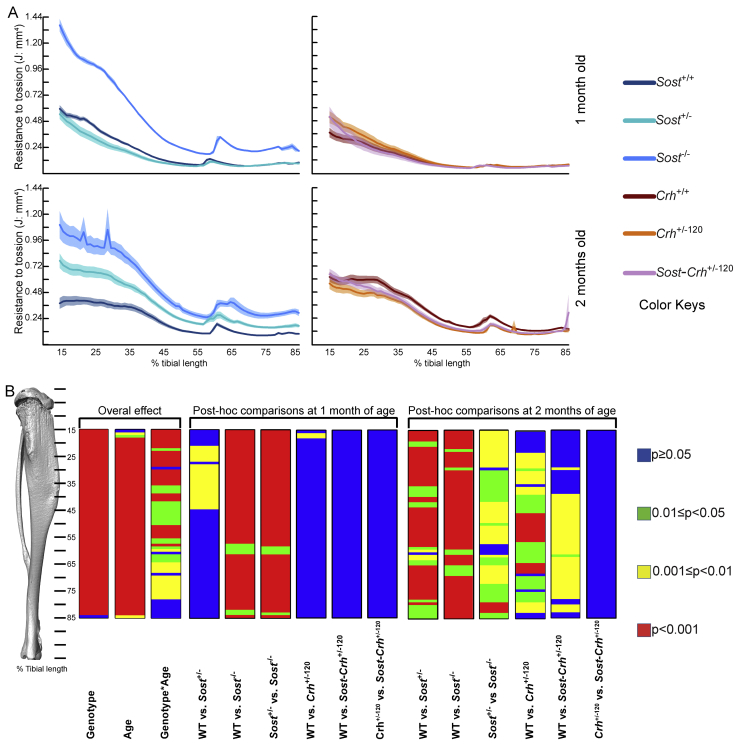
*Sost* haploinsufficiency does not rescue reduced resistance to torsion (J) induced by excessive glucocorticoids (GCs). **A:** J in *Sost* wild-type (WT) (dark blue; *Sost*^+/+^ WT), *Sost* heterozygous knockout (KO) (light blue; *Sost*^+/−^), *Sost* homozygous KO (turquoise; *Sost*^−/−^), *Crh* WT (dark red; *Crh*^+/+^), *Crh* heterozygous (orange; *Crh*^+/−120^), and double *Sost*-*Crh* heterozygous (plum; *Sost*-*Crh*^+/−120^) mice at 1 and 2 months of age. Lines represent the means; shading, the SEM. **B:** Graphic heatmap representation of statistical significance of the effect of genotype, age, their interactions as well as *post hoc* analysis along a 15% to 85% portion of the whole tibia length, excluding proximal and distal metaphyseal bone. *n* = 8 mice per group.

**Supplemental Figure S2:**
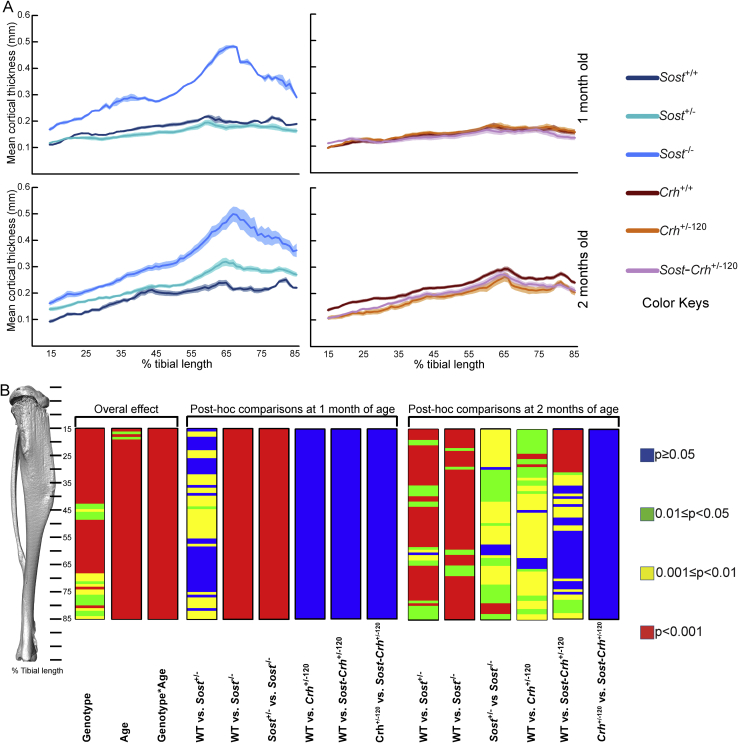
*Sost* haploinsufficiency does not rescue reduced mean cortical thickness (Cs.Th) induced by excessive glucocorticoids (GCs). **A:** Torsion (J) in *Sost* wild-type (WT) (dark blue; *Sost*^+/+^ WT), *Sost* heterozygous knockout (KO) (light blue; *Sost*^+/−^), *Sost* homozygous KO (turquoise; *Sost*^−/−^), *Crh* WT (dark red; *Crh*^+/+^), *Crh* heterozygous (orange; *Crh*^+/−120^), and double *Sost*-*Crh* heterozygous (plum; *Sost*-*Crh*^+/−120^) mice at 1 and 2 months of age. Lines represent the means; shading, the SEM. **B:** Graphic heatmap representation of statistical significance of the effect of genotype, age, their interactions as well as *post hoc* analysis along a 15% to 85% portion of the whole tibia length, excluding proximal and distal metaphyseal bone. *n* = 8 mice per group.

**Supplemental Figure S3:**
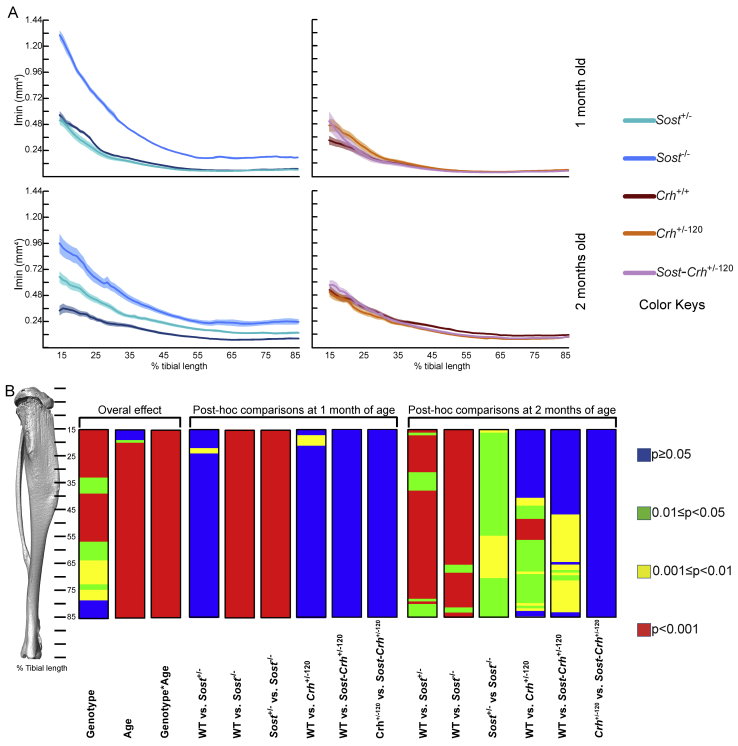
*Sost* haploinsufficiency does not rescue reduced area around minor axis (I_min_) induced by excessive glucocorticoids (GCs). **A:** I_min_ in *Sost* wilt-type (WT) (dark blue; *Sost*^+/+^ WT), *Sost* heterozygous knockout (KO) (light blue; *Sost*^+/−^), *Sost* homozygous KO (turquoise; *Sost*^−/−^), *Crh* WT (dark red; *Crh*^+/+^), *Crh* heterozygous (orange; *Crh*^+/−120^), and double *Sost*-*Crh* heterozygous (plum; *Sost*-*Crh*^+/−120^) mice at 1 and 2 months of age. Lines represent the means; shading, the SEM. **B:** Graphic heatmap representation of statistical significance of the effect of genotype, age, their interactions as well as *post hoc* analysis along a 15% to 85% portion of the whole tibia length, excluding proximal and distal metaphyseal bone. *n* = 8 mice per group.

**Supplemental Figure S4:**
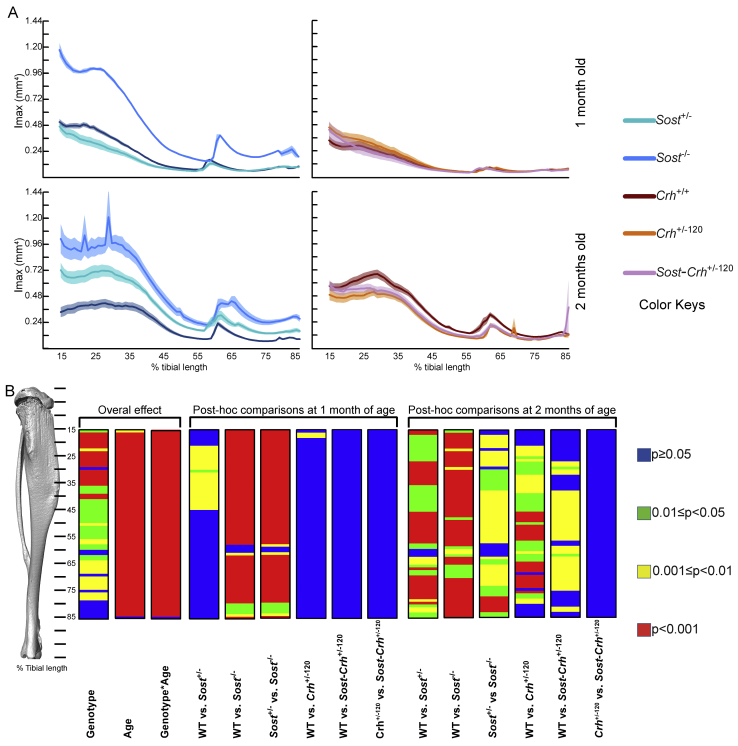
*Sost* haploinsufficiency does not rescue reduced area around major axis (I_max_) induced by excessive glucocorticoids (GCs). **A:** I_max_ in *Sost* wild-type (WT) (dark blue; *Sost*^+/+^ WT), *Sost* heterozygous knockout (KO) (light blue; *Sost*^+/−^), *Sost* homozygous KO (turquoise; *Sost*^−/−^), *Crh* WT (dark red; *Crh*^+/+^), *Crh* heterozygous (orange; *Crh*^+/−120^), and double *Sost*-*Crh* heterozygous (plum; *Sost*-*Crh*^+/−120^) mice at 1 and 2 months of age. Lines represent the means; shading, the SEM. **B:** Graphic heatmap representation of statistical significance of the effect of genotype, age, their interactions as well as *post hoc* analysis along a 15% to 85% portion of the whole tibia length, excluding proximal and distal metaphyseal bone. *n* = 8 mice per group.

**Supplemental Figure S5:**
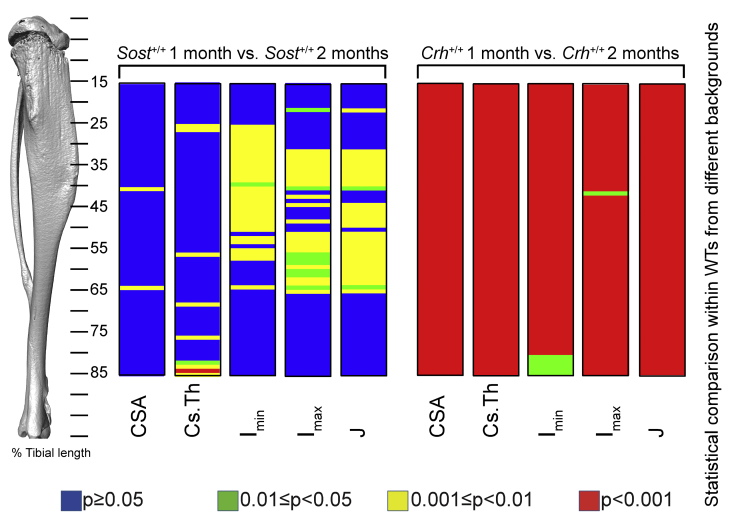
Graphic heatmap representation of statistical comparison within wild-type (WT) mice from different backgrounds. *Sost*^+/+^ and *Crh*^+/+^ at 1 month of age compared with *Sost*^+/+^ and *Crh*^+/+^ mice at 2 months of age for cross-sectional area (CSA), mean cortical thickness (Cs.Th), second area around minor axis (I_min_), second area around major axis (I_max_), and resistance to torsion (J). *n* = 8 mice per group.

Analysis of mice with excessive endogenous GCs at 1 month of age failed to find any significant differences in cortical CSA at any location along the tibia in WT *Crh* (*Crh*^+/+^ WT), heterozygous *Crh* (*Crh*^+/−120^), or mice heterozygous for both *Crh* and *Sost* (*Sost*-*Crh*^+/−120^) (Figure 1). In contrast, in mice aged 2 months, CSA was, as expected, significantly lower in *Crh*^+/−120^ mice compared with *Crh*^+/+^ WT mice and also lower in *Sost*-*Crh*^+/−120^ mice compared with *Crh*^+/+^ mice between approximately 25% and 85% of the tibial length. Intriguingly, no significant rescue in the reduction of CSA was observed at any location in tibia from *Sost*-*Crh*^+/−120^ mice compared with *Crh*^+/−120^ mice. Evaluation of torsion, a measure of predicted resistance to torsion, showed identical trends, indicating that *Sost* haploinsufficiency also failed to rescue GC-induced decreases in the tibia's architectural strength (Supplemental Figure S1). Further interrogation of cortical bone revealed that mean cortical cross-sectional thickness (Supplemental Figure S2), second area around minor axis (Supplemental Figure S3), and around major axis (Supplemental Figure S4) also indicated that *Sost* haploinsufficiency failed to rescue *Crh*^+/−120^–induced compromise in cortical bone mass and architecture. Our evaluation of age-related changes in the WT mice of both *Sost*^−/−^ (C57BL/6NTac/C57BL/6J) and *Crh*^+/−120^ (C57BL/6J/C3H/HeH) backgrounds revealed greater growth kinetics within the latter, between 1 and 2 months of age, suggesting that there was ample scope for any effects of the combined genetic manipulation to be realized (Supplemental Figure S5).

**Figure 2:**
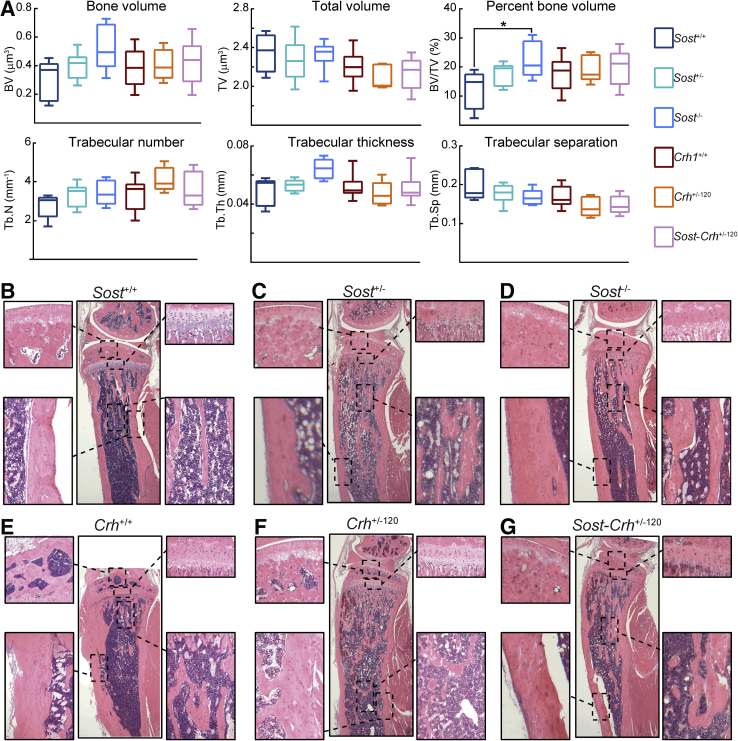
Trabecular bone phenotype of *Sost* wild-type (WT) (*Sost*^+/+^), *Sost* heterozygous knockout (KO) (*Sost*^+/−^), *Sost* homozygous KO (*Sost*^−/−^), *Crh* WT (*Crh*^+/+^), *Crh* heterozygous (*Crh*^+/−120^), and double *Sost*-*Crh* heterozygous (*Sost*-*Crh*^+/−120^) mice at 2 months of age. **A:***Ex vivo* high-resolution analyses of the proximal metaphyseal tibia to determine trabecular bone volume (BV), trabecular total volume (TV), percentage of bone volume (BV/TV), trabecular number (Tb.N), trabecular thickness (Tb.Th), and trabecular separation (Tb.Sp). **B–G:** Representative hematoxylin and eosin–stained sections of tibia at 2 months of age. **B:***Sost* WT (*Sost*^+/+^) mice. **C:***Sost* heterozygous KO (*Sost*^+/−^) mice. **D:***Sost* homozygous KO (*Sost*^−/−^) mice. **E:***Crh* WT (*Crh*^+/+^) mice. **F:***Crh* heterozygous (*Crh*^+/−120^) mice. **G:** Double *Sost*-*Crh* heterozygous (*Sost*-*Crh*^+/−120^) with corresponding higher magnification demonstrating the structural differences between groups. Data are expressed as means ± SEM. *n* = 8 mice per group. ^∗^*P* < 0.05. Original magnification: ×2 (**B**–**G**, main images); ×10 (**B**–**G**, enlarged images)

In addition to microCT analysis of cortical bone, tibial trabecular bone was analyzed. Percentage of bone volume [trabecular bone volume (BV)/trabecular total volume (TV)] was significantly greater in mice lacking both copies of *Sost* (*Sost*^−/−^), whereas a trend that did not reach levels of statistical significance was observed for enhanced BV/TV in mice lacking a single copy (*Sost*^+/−^) (Figure 2A). This elevation of BV/TV in *Sost*^−/−^ mice was linked with enhanced BV, trabecular number, and thickness, and lower trabecular separation; neither of these variables alone reached statistical significance (Figure 2A). The analysis also showed a trend for lower trabecular BV/TV in mice with excess GCs (*Crh*^+/−120^) and no significant modification of BV/TV in *Sost-Crh*^+/−120^ mice (Figure 2A). In agreement with the microCT data, detailed histologic evaluation of tibia structure revealed thicker cortices in tibiae from mice lacking either one (*Sost*^+/−^) or both (*Sost*^−/−^) copies of functional *Sost* compared with tibia from *Sost*^+/+^ WT mice (Figure 2, B–G). Thinner cortices were also observed in *Crh*^+/−120^ mutant mice compared with tibiae from their *Crh*^+/+^ WT littermates. In agreement with three-dimensional quantification, tibiae from mice harboring both *Sost*^+/−^ and *Crh*^+/−120^ (*Sost*-*Crh*^+/−120^) were similar to *Crh*^+/−120^ mice, indicating a failure of *Sost*^+/−^ to rescue the effects of GC excess on bone mass (Figure 2, E–G). Together, the detailed comparison of bone by both histology and microCT revealed at 2 months of age that i) both hetero- and homozygous *Sost*-deficient mice exhibited significantly greater bone mass and predicted strength, ii) heterozygous *Crh* mutation led to a compromised bone mass and strength and, iii) *Sost* haploinsufficiency fails to rescue the compromised bone mass produced by excess GCs.

### Combined *Sost* Haploinsufficiency and *Crh*^+/−120^ Related GC Excess Provoke Peracute Lethal Cardiac Tamponade

In the studies that examined potential rescue of excess GC-related low bone mass by deletion of a single *Sost* allele, sudden, unprovoked, nonconvulsive death of approximately 10% of total *Sost*-*Crh*^+/−^ offspring was observed between 1 to 2 months of age. No such incidences occurred in either *Crh*^+/−120^, *Sost*^−/−^ mice or their respective WT littermates. To identify the cause of this sudden death, a comprehensive histopathologic examination of multiple tissues from all mouse strains was performed hematoxylin and eosin staining, which was scored with a semiquantitative grading system. Examination of the brain, kidney, liver, lung, and spleen showed no signs of overt pathology in *Sost*^+/+^ WT, *Sost*^+/−^, *Sost*^−/−^, *Crh*^+/+^ WT or *Crh*^+/−120^, or *Sost*-*Crh*^+/−120^ mice in which a propensity for sudden death was observed.

**Figure 3:**
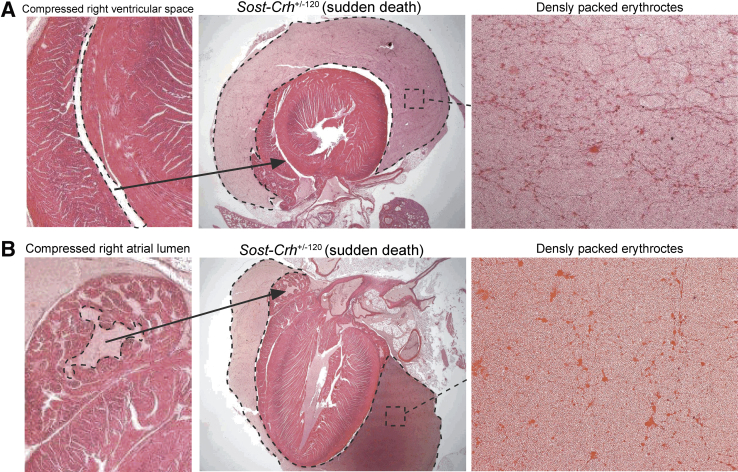
*Sost* haploinsufficiency together with excessive glucocorticoids (GCs) leads to peracute lethal cardiac tamponade. Two representative cases are displayed. **A:** Markedly expanded pericardial space (**dashed line** in **middle panel**) with a large number of densely packed, homogenously distributed erythrocytes and compressed right ventricular lumen. **B:** Hemopericardium (**dashed line** in **middle panel**) containing erythrocytes and a compressed right atrial lumen. Original magnification: ×2 (**A** and **B**, **left panel**); ×1.25 (**A** and **B**, **middle panel**); ×10 (**A** and **B**, **right panel**).

Intriguingly, evaluation of heart tissue in *Sost*-*Crh*^+/−120^ mice with sudden death revealed all to have hemopericardium with a markedly expanded pericardial space that contained a large number of densely packed, homogenously distributed erythrocytes and compression of the right ventricular (Figure 3A) and right atrial lumen (Figure 3B) in all mice that experienced sudden death. Therefore, the cause of sudden death in these animals was considered to be due to peracute hemopericardium that led to cardiac tamponade. Hearts were examined at multiple step levels through the tissue to try to ascertain the origin of the hemorrhage; however, none could be identified. No significant findings were observed in the hearts of the remainder of the *Sost*-*Crh*^+/−120^ mice, or in any of the *Sost*^+/+^ WT, *Sost*^+/−^, *Sost*^−/−^, *Crh*^+/+^ WT, and *Crh*^+/−120^ mice.

## Discussion

Osteopenia because of excess GCs mainly involves a decrease in bone formation, secondary to the effects on the activity and viability of osteoblasts and osteocytes, which ultimately leads to an impairment in bone strength. One of the main mechanisms by which GCs achieve such deleterious outcome in bone is by antagonizing Wnt/β-catenin signaling. Sclerostin, the product of the *Sost* gene, is the main negative regulator of the Wnt pathway in the skeleton. Thus, sclerostin normally acts to suppress the proformative and antiresorptive role of the Wnt signaling pathway. Herein, the hypothesis that the partial inactivation of sclerostin function by genetic manipulation will be capable of rescuing the low bone mass induced by high endogenous GC levels was tested.

In contrast, it was found instead that *Sost* haploinsufficiency was not capable of rescuing the low bone mass induced by high GC. These findings do not agree with previous studies which reported that transient antisclerostin antibody treatment rescued GC-induced low bone mass.^41^, ^42^ It is possible that these dissimilar observations arise due solely to differences in experimental design and analysis. These studies undertook a nonbiased analysis of almost the entire tibial cortex adjusting for GC-induced divergence in bone length and thus ensure comparisons are made at precisely matched anatomic locations and volumes. Although Marenzana et al^42^ reported small, significant protective effects of antisclerostin antibody treatment against an exogenous GC-induced reduction in cortical bone volume at the femur mid-shaft, they did not find similar rescue of GC-induced reduction in bone length. The methods used, however, made no apparent correction for this growth retardation; thus, measurement of equivalent bone volumes was not ensured.^42^ An alternative explanation is that our studies encompass the developmental effect of excessive GCs and *Sost* haploinsufficiency, and it remains possible that this may diverge from the outcome of transient antisclerostin antibody treatment of adult mice that have induced GC excess.^41^, ^42^

Sclerostin has long been considered an effective target for treating osteoporosis. The most recent phase 3 clinical trial of romosozumab (an antisclerostin antibody), to our knowledge, was however hampered by unforeseen, increased adverse cardiovascular risk,^50^ resulting in refusal to approve this treatment by the Food and Drug Administration. The finding that a combination of high endogenous GC levels together with *Sost* haploinsufficiency results in sudden death with histopathologic, marked hemopericardium, is therefore intriguing. The pericardial space in these mice was found to be markedly enlarged by densely packed erythrocytes with compression of the heart lumen, consistent with sudden death from cardiac tamponade.

To our knowledge, cardiac tamponade secondary to spontaneous hemopericardium has not been reported before in mice. In other species, causes of spontaneous hemopericardium occur because of rupture of the intra-pericardial portion of the aorta or pulmonary artery, rupture of a coronary vessel, cardiac rupture after an acute myocardial infarct or rupture of an atrium secondary to atrial thrombosis, blood dyscrasias, and neoplasia. The histologic examinations, however, did not localize the site of rupture/hemorrhage or reveal an overt underlying pathology. Microscopic examination of the heart and major blood vessels in both affected and unaffected animals also failed to disclose any possible predisposing lesions such as aneurysm, congenital blood vessel anomaly, or cardiac disease.

Several possible explanations may clarify the underpinning mechanisms whereby sclerostin exerts a regulatory role in the cardiovascular system. Although sclerostin is reported to be expressed in the aorta,^51^, ^52^, ^53^ neither *Sost* KO mice in this study or others nor human patients with sclerosteosis or van Buchem's disease due to mutations in the *SOST* gene exhibit any greater risk of cardiovascular complications.^54^, ^55^, ^56^, ^57^ Furthermore, a case study by van Lierop et al^58^ reported that GC treatment does not lead to adverse cardiovascular effects in a van Buchem high bone mass patient with mutation in the *SOST* gene. In addition, Sato et al^59^ reported that *Sost* homozygous KO mice treated with GCs maintain structural and material mechanical properties despite increased osteocyte apoptosis and that no cardiovascular complications were reported. It is possible that differences in our findings to those reported by aforementioned studies can be explained by the fact that the excess GCs present in *Crh*^+/−120^ mice during developmental and later life-course may affect multiple organs, including the cardiovascular system.

Other studies have also reported up-regulation of sclerostin in foci of vascular and valvular calcification.^60^, ^61^, ^62^, ^63^ Our detailed histopathologic examinations were not able to find any signs of calcification in any mice in this study. This agrees with other animal studies showing that sclerostin is unlikely to be involved in vascular mineralization.^52^, ^64^ No evidence suggests that sclerostin plays a causal role in cardiovascular disease, rather recent studies have reported an association between sclerostin levels and cardiovascular disorders.^65^, ^66^, ^67^, ^68^ The precise function of sclerostin in the regulation of the cardiovascular system remains to be defined.

A limitation in our studies is the lack of any measured levels of circulatory sclerostin; thus, it remains to be verified that the *Sost* genetic haploinsufficiency in our studies is necessarily reflected in a change in expression levels. The middle level bone phenotype observed in the *Sost* haploinsufficient mice (either in mice with or without the *Crh*^+/−120^ mutation) in which true heterozygosity was also confirmed by genotyping makes it highly likely that sclerostin levels are modified accordingly. The levels might be lower in the *Sost*-*Crh*^+/−120^ mice than in *Crh*^+/−120^ mice. Another limitation is that whether antisclerostin antibody–mediated blockade of sclerostin function is equivalent to genetic insufficiency cannot be predicted.

Nonetheless, the critical manifestation of *Sost*-deficiency combined with GC excess in our study is sporadic, sudden, unprovoked, and nonconvulsive death linked to peracute hemopericardium and cardiac tamponade. Tamponade has been linked to many conditions, including cancer, uremia, hypothyroidism, and pericarditis, where enlargement of pericardium arises gradually.^69^, ^70^, ^71^, ^72^ No evidence for such conditions was found in this study. The rapid hemopericardium-related tamponade observed is more consistent with myocardial rupture, but this was not found on detailed pathologic examination using multiple step levels sectioned throughout the heart. The cause of this tamponade and death in some *Sost*-*Crh1*^+/−120^ mice remains unexplained; this is confounded by a lack of any abnormal preceding changes evident in their living equivalents; no predisposing lesions, such as aneurysm, focal thinning, or disruption of the myocardium or coronary vessels were observed histologically in the unaffected animals. It is tempting to speculate on the likely mechanisms. Experiments that explore whether unrestricted Wnt signaling, due to *Sost* deficiency, interacts with regulators of inflammatory processes, as seen in circumstances such as GC excess, may explain such serious adverse cardiovascular events. These findings that use mouse preclinical models, therefore, prompt further studies aimed at deciphering their clinical significance and the underpinning molecular mechanisms by which GC-induced osteopenia might be better-targeted without risk of cardiovascular side effects.

## Supplemental Data

Data Profile
